# Core-periphery structure in sectoral international trade networks: A new approach to an old theory

**DOI:** 10.1371/journal.pone.0229547

**Published:** 2020-04-02

**Authors:** Olivera Kostoska, Sonja Mitikj, Petar Jovanovski, Ljupco Kocarev

**Affiliations:** 1 Faculty of Economics-Prilep, “St. Kliment Ohridski” University, Bitola, North Macedonia; 2 Macedonian Academy of Sciences and Arts, Skopje, North Macedonia; 3 Faculty of Computer Science and Engineering, “Ss. Cyril and Methodius” University, Skopje, North Macedonia; Tohoku University, JAPAN

## Abstract

The research on core-periphery structure of global trade from a complex-network perspective has shown that the world system is hierarchically organized into blocks and that countries play different roles in the world economy. Yet, little attention has been paid to investigating whether the sectoral international trade networks conform to a core-periphery structure, hence what is the role of different levels of processing in creating and maintaining structural inequality. This issue is of particular importance given the contemporary focus upon global production networks and reshaping of the international division of labor. With this in mind, we propose a model (LARDEG) from network science to reexamine old theories in economics, such as core-periphery structures in sectoral international trade networks and test whether the global value chains have changed structural positions in terms of the level of processing. The economic background of our model permitting a more accurate sorting of countries into structural positions and the general stability of results have provided for a more solid measurements than has hereto been possible. Our algorithm naturally produces networks with hierarchically nested block structure obtained from an iterative decomposition of the network periphery such that each block represents a vertex set of a maximal size sub-graph existing at different levels. The results not only lend support to the previous hierarchical model of the world-system (core, semi-periphery, and periphery) but also find that, depending on particular industry, the number of analytically identifiable blocks could be more than three. We show that ‘size effect’ is the one that prevails for core block membership at the first hierarchical level, while the GNI per capita is a much poorer proxy for the world-system status. Moreover, the patterns of blocks we label as the second- or third-level ‘core’ are strongly dependent on distance and geographical proximity. Overall, the various configurations of asymmetrical trade patterns between our blocks and the remarkably stable position of core countries at the top of structure clearly indicate that the rise of global production networks has actually restored a huge and unequal international division of labor splitting the world into ‘headquarter’ and ‘factory’ economies.

## Introduction

The ‘world-system’ and ‘dependency’ theory (also known as the core-periphery theory) assert that the structure of the world economy produces international inequality. Technological and other patterns of dependency confine the sectors in developing economies to less sophisticated forms than those prevailing in core nations [1, 2]. The structure of international trade is therefore hierarchically organized into blocks (core, semi-periphery, and periphery) in line with the extent to which they are drawn into the core or periphery production processes. Put differently, developing countries are poor as a result of their trade specialization and embeddedness in the global trading system, in which the core countries settle on a diverse set of knowledge-intensive and value-added products (required by all parts of the world economy), while the peripheral developing countries specialize in exports of simple resource and labor exploiting products to higher blocks of the hierarchy [3–5]. Global economy, however, changes rapidly, and the same is true for the underlying structural interdependencies of the countries in the global system. Global production networks or global value chains (GVCs) can provide a great opportunity for developing countries to absorb knowledge and technology in all their agricultural, manufacturing, and service production [6], as well as to take part in global markets without having to develop complete products or value chains [7, 8]. However, even when developing economies manage to get into the global value chains, they may still face the risk of persisting in low value added positions, with narrow learning and upgrading possibilities [9]. A critical question is then, is traditional core-periphery structure of international trade still alive? To what extent changes in the international division of labor have actually led to upward (or downward) mobility of countries in the core-periphery structure? Is there a real benefit of GVCs participation for developing countries in terms of long-term impact on capacity building and sustainability of the local industrial base or it simply results in different forms of dependence and reproduction of global inequality [10]?

This paper contributes to this debate by applying methods from a network science to re-examine the ideas about the core-periphery concept of international trade from a different standpoint. The number of articles addressing the international-trade issues from a complex-network perspective [11–17], and especially those investigating the polarized (core-periphery) trade structure [18–29] has been growing over the last decades. However, very few studies (to the best of our knowledge, almost none in recent years) focus specifically on core-periphery structure in sectoral international trade networks. Global production networks make a connection between spatially dispersed activities into a single sector (industry) and help understand the shifting patterns of trade and production. Hence, to properly assess the structural inequality and to evaluate the time-related mobility of particular countries within the structure, one needs to take into account different levels of processing with regard to goods the countries are able to produce and export. This paper resolves these challenges of interpretation by evaluating the extent to which sectoral international trade networks conform to a core-periphery structure over the period 2000-2016. Moreover, by taking recent points in time into analysis (including the post-crisis 2008-2009 period), the article provides results that bear on a number of issues comprising the crisis-related structural changes in the world trade, as well as the stability and dynamism in the contemporary global division of labor that were beyond the scope of the previous quantitative research [20–22]. From a methodological point of view, this work contributes to the existing literature by proposing a model (we label as LARDEG) to identify the (meso-scale) core-periphery structures in distinctive industries (as binary directed networks). More specifically, the core-periphery structure of trade networks here will be examined by comparing the results of our proposed algorithm to those obtained from the existing models, that is (1) the deterministic block model for weighted directed networks that uses regular equivalence implemented with REGE algorithm [21]; (2) the SBM for weighted (directed) network [30, 31]; (3) the continuous multiplicative core-periphery model for for weighted directed networks, implemented with MINRES/SVD algorithm [32]. The clear economic grounds of our model, as well as the general stability and analytical interpretability of results allow us to come up with a more precise allocation of countries into structural positions. Our mechanism gives rise to networks with nested core-periphery structure, i.e. blocks obtained from a further decomposition of the network periphery such that each block embodies a vertex set of a maximal size sub-graph existing at different hierarchical levels. The algorithm is parameter free so that the blocks are detected without the need for any predefined parameters. Moreover, it is computationally efficient (in fact, the algorithm is optimal) and can be used for partitioning the vertex sets of large-scale graphs. The general patterns lend credibility to the Wallerstein’s [4] hierarchical model of the world economy (core, semi-periphery, and periphery). Nevertheless, our results suggest that, depending on particular sector, the number of blocks could be more than three—they range from 4 to 9 blocks that may either correspond to some blocks of Smith and White’s [21] five strata terminology or need to receive further investigation. Of particular importance in the context of the discussion presented in the trade literature is the reason behind these groups and subgroups of nodes existing at different levels. To this end, we provide strong empirical evidence that helps to determine the significance of the so-called ‘size effect’ and/or ‘income effect’, as well as the role of geographical distance (together with regional trade agreements) and associated trade costs in selecting the trading partners and producing a hierarchical structure at different network levels. Network analysis conducted here permits a careful examination of the trade relationships within and between blocks. The results are entirely consistent with the world-system/dependency hypothesis that high-technology manufactures are likely to be exported from the core to periphery and that the flows coming from the periphery to core structures of these industries consistently show negligible results. Moreover, the position of the core countries is outstandingly stable at the top of the structure, with a leading role played by the major European countries, the United States and the emerging market of China. Even the financial crisis of 2008 did not change the core positions in 2009 and that would also imply that the structure of GVC networks is resilient. Overall, our results demonstrate clearly that the rise of GVCs, led by a select group of powerful countries, has reestablished an enormous and unequal global division of labor.

## Materials and methods

### Data

International trade network is defined as the graph of import/export relationships between countries in a given year, *G* = (*V*, *E*) where *n* = |*V*| is the number of countries that constitute the vertices (nodes) of the graph, and *m* = |*E*| is the number of existing directed trade links (directed edges, or arcs). Before going any further, let us define **W** as *n* × *n* matrix of the corresponding weighted directed ITN, where *w*_*ij*_ stands for an element of **W** representing a positive-valued export flow that goes from the country of origin *i* to the point of destination *j* (and zero if the corresponding trade flow is zero). We can also define a binary matrix **A** as *n* × *n* matrix such that *a*_*ij*_ = 1 if and only if *w*_*ij*_ > 0, and zero otherwise. Consequently, both representations of the international trade network (binary directed and weighted directed) are constructed for the purpose of analysis conducted here. The binary directed representation provides information for the presence or absence of trade partnerships, while the weighted directed representation adds to the binary structure some additional information about each link of the graph (e.g. positive weights that usually indicate the strength of the relationship).

This paper employs data on the value (in U.S. dollars) of bilateral trade for the years 2000, 2005, 2009, 2012, and 2016 available from UN Comtrade. The goods are classified according to the Standard International Trade Classification (SITC), providing for international comparisons of primary products (SITC 0-4) and manufactured goods (SITC 5-8). The SITC has actually been developed by the United Nations with the intention of reflecting not only the material properties, but also the processing stage, technological changes and uses of products [33]. The classification is hierarchically organized into codes (from very general, one-digit codes to the extremely specific, five-digit codes). Put differently, the five-digit SITC classification offers a more detailed list of merchandise. Alternatively, the broad, one-digit numbers provide an easier way to distinguish between raw materials and finished products, but there is no homogeneity in these highly aggregated categories (for example, general headings like “crude materials, inedible, except fuels” or “manufactured goods classified chiefly by material” consist of a wide variety of articles, ranging from products that require minimal processing to those created in high-technology environments). In order to examine more homogenous structures, the two-digit level (63 industries) is used in this study. Whilst these still may hold some internal heterogeneity, we have opted for this level of aggregation because measurement errors and misclassifications turn out to be even more problematic when applying more specific SITC categories (for the advantages of this classification and the selection of appropriate level of aggregation, see also [34]). What is more, using the two-digit over more specific categories confines the series of possible matrices from thousands to sixty-three. This means that 63 distinctive networks are constructed for one year (or 630, both binary and weighted directed networks for each of the 63 sectors at the five time points).

In line with Hartmann et al. [27], we lessen the noise hailing from underreporting and from variations in size of countries and products by applying several filters. First, as a partial control for the huge differences in country size, the analysis includes only those countries with populations of over one million. For a variety of reasons, several countries failed to report data for each year. In this case, the number of countries reduces to 133 for 2000, 129 for 2005, 130 for 2009, 129 for 2012, and 124 for 2016. The final analysis, however, examines only those networks of trade between countries in which data is available at all time points. Hence, the network reduces to 109 vertices, viz. 109 countries making up an average of 86.79% of the world population and an average of 95.08% of the global trade value of goods exported throughout the world over the five-year period. Although these countries account for 95.08% of the world exports, a portion of transactions ends up in countries that have been removed, or not represented by vertices in the network. This reduces the network coverage to another 86.24% of the world exports for the five-year period. Given that the analysis does not include information on the ninth sector (commodities and transactions not classified elsewhere in the SITC), the network coverage declines further to 82.37% of the global exports. Finally, in order to avoid additional distortions, the network analysis excludes all yearly trade flows valued at less than or equal to US $10,000. It is noteworthy that filters with different thresholds were applied (e.g. US $20,000 or US $5,000), yet the one with US $10,000 has proved the most reliable in keeping the balance between the network coverage of world exports and the link retention. While the number of arcs slightly decreases, the network still covers around 82.37% of world exports.

### Methodology: Existing models and our approach

#### Existing models

Networks can be represented by a mixture of local, global, and intermediate-scale (mesoscale) perspectives. The algorithmic identification of mesoscale network structures provides the means to discover distinctive attributes that are not clearly visible either at local level of vertices and arcs or at global level of summary statistics. The mesoscopic network structure known as core-periphery structure, in its simplest form, brings up a partition of a network into two groups of vertices (core and periphery). The concept of the network core usually refers to a central and densely connected set of network vertices, while the periphery stands for a sparsely connected and frequently non-central set of nodes that are linked to the core. Hence, a certain vertex belongs to the core if and only if it is well connected both to other core and peripheral vertices. The core-periphery structure of networks have been examined by two quantitative approaches, both originating from the realm of social networks. The first refers to the stochastic block modeling. A stochastic block-model (SBM) is a network model that presumes a latent clustering, called stochastic equivalence, of the vertices into non-overlapping groups, such that the distribution of the edges is fully determined by the clusters. SBMs are used to detect prevailing mesoscale structures without assuming any specific structure a priori. Yet, they have not been used to model trade networks so far, and are rarely employed in the analysis of core-periphery structures (an exception is the work of Zhang and Thill [30]). Historically, SBMs are introduced by Holland et al. [35] as an extension of two other types of equivalence: structural and regular. It is worth mentioning that Smith and White [21] used regular equivalence (implemented with REGE algorithm) to analyze the presence of five blocks or strata in the world-economy, the so-called: core, strong semi-periphery, weak semi-periphery, strong periphery, and weak periphery. However, since Borgatti and Everett [36] formally defined the notion of a core-periphery structure in 2000, it is still an open question how these five mesoscale strata are defined and/or related to the formal core-periphery definition.

When it comes to the second approach, Borgatti and Everett [36], in addition to formally defining the core-periphery structure, have also developed algorithms for identifying discrete and continuous versions of core-periphery structure. This work triggered a plethora of different models for core-periphery detection, all based on the definition of various quality functions and their optimization over certain discrete or continuous sets of vectors. In this setting, each node is assigned a measure of “coreness”, such that a larger value points to a greater coreness. In particular, for the continuous multiplicative core-periphery model, which uses MINRES algorithm, as discussed, for instance, by Boyd et al. [32], the measure of coreness is precisely the centrality measure of Bonacich [37]. Moreover, Boyd et al. [32] suggested MINRES/SVD (minimum residual singular value) algorithm for addressing core-periphery structures in directed networks: each node in the network receives two indices, an “in-coreness” and an “out-coreness” (for a recent review of the various core-periphery models, see also Rombach et al. [38]).

#### Partitioning the vertex set

Giatsidis et al. [39] have recently proposed novel metrics to measure community detection in directed networks by extending the well-known graph-theoretic notion of *k*-cores to directed graphs. Here we adopt the concept of (*k*, *l*)-cores for directed graphs and show that it can also be extended for detecting core-periphery structures in binary directed networks. Moreover, the algorithm for finding a single (*k*, *l*)-core in directed networks, has been recursively applied for the whole vertex set, resulting in a unique set of subsets on which the vertex set is partitioned. We call the resulting partitioned set, the LARDEG (for largest degeneracy) model. Therefore, the model LARDEG addresses partitioning the vertex set of a graph by graph-theoretic notion of (*k*, *l*)-cores for binary directed graphs, which is considered as a measure of the robustness of a sub-graph under degeneracy [39]. Degeneracy is a graph measure (also known as the *k*-core number, width, and linkage) that is related to the coloring number. Recall, for undirected graphs, a *k*-core of a graph *G* is a maximal connected subgraph of *G* in which all vertices have degree at least *k*. If a non-empty *k*-core exists, then the degeneracy of *G* is the largest *k* for which *G* has a *k*-core. For directed graphs, degeneracy of a digraph *G* is introduced by Giatsidis et al. [39]. The min-in-degree and the min-out-degree of a digraph *G* are defined as follows:

$$\begin{array}{ccc} \delta^{in}\left(G\right) & = & \min\{d_{i}^{in},i\in V\} \end{array}$$

$$\begin{array}{ccc} \delta^{out}\left(G\right) & = & \min\{d_{i}^{out},i\in V\} \end{array}$$

respectively, where

$$\begin{array}{ccc} d_{i}^{in} & = & \sum_{j}a_{ji} \end{array}$$ (1)

$$\begin{array}{ccc} d_{i}^{out} & = & \sum_{j}a_{ij} \end{array}$$ (2)

Given two positive integers *k*, *l* and a digraph *G*, (*k*, *l*)-core of *G* is a maximal size sub-digraph *F* of *G* where *δ*^*in*^(*F*)≥*k* and *δ*^*out*^(*F*)≥*l*; if no such digraph exists then the (*k*, *l*)-core of *G* is the empty digraph. The degeneracy of a digraph *G* is defined as follows:

$$\begin{array}{c} \delta^{⋆}=\frac{1}{2}\max\{\delta^{in}\left(H\right)+\delta^{out}\left(H\right)|H\subseteq G\} \end{array}$$

Therefore, *δ*^⋆^ returns the maximum *r* (for some pair (*k*, *l*) for which *k* + *l* = 2*r*) such that *G* contains a non-empty (*k*, *l*)-core.

Let *G* = (*V*, *E*) be a directed graph with *n* = |*V*| vertices and *m* = |*E*| links. Assume *G* contains a (*k*_1_, *l*_1_)-core, that is, a maximal size sub-digraph and let *V*_1_ ⊂ *V* be its vertex set. In this case, the vertex set *V* is a union of two nonempty disjoint subsets: (*k*_1_, *l*_1_)-core *V*_1_ and the set *U*_1_ = *V*\*V*_1_. Moreover, the graph *G* can be represented as a union of three induced sub-graphs: sub-graph $G_{1}^{V}$ with the vertex set *V*_1_, sub-graph $G_{1}^{U}$ with the vertex set *U*_1_, and bipartite sub-graph with vertex sets *V*_1_ and *U*_1_. We perform the procedure again for $G_{1}^{U}$: let (*k*_2_, *l*_2_) be a maximal size sub-digraph of $G_{1}^{U}$. In this case, the vertex set *U*_1_ can be represented as union of two nonempty disjoint subsets: (*k*_2_, *l*_2_)-core *V*_2_ and the set *U*_2_ = *U*_1_\*V*_2_. The same procedure is repeated until for some finite *r*, *V*_*r*_ ≠ ∅, while *U*_*r*_ = ∅. Therefore, the vertex set *V* is partitioned into *r* disjoint blocks *V* = *V*_1_∪*V*_2_…∪*V*_*r*_ such that *k*_*i*_ ≥ *k*_*i*+1_, *l*_*i*_ ≥ *l*_*i*+1_ and *k*_*i*_ + *l*_*i*_ > *k*_*i*+1_ + *l*_*i*+1_. This hierarchically nested block structure will be analyzed by considering pairs $\left(V_{i},U_{i}=\cup_{k=i+1}^{r}V_{k}\right)$, for *i* = 1, …, *r*−1, such that the pair (*V*_*i*_, *U*_*i*_) will be labelled as *i*-level structure (for the methodologies to observe nestedness in complex networks, see [40]; for a recent attempt to examine nestedness using a multi-layer representation of the worldwide trade network, see also [41]).

#### Null models

In the context of trade networks, the network null hypothesis (null model) addresses an expectation that the trade relationships are drawn from a single distribution, so that any patterns in the adjacency matrix data arise only from random sampling processes. The alternative hypothesis is that patterns in the data are not the result of a random variation generated by the null hypothesis. Here we consider three null models for examining the nested core-periphery structure in trade networks: (1) not preserving the nodes’ in- and out-degree, and assuming that all nodes have the same probability to be involved in trade relationships; (2) preserving, on average, the nodes’ in- and out-degree (the trade interaction probability is proportional to degree); and (3) preserving exactly the individual nodes’ in- and out-degree.

The null hypothesis varies depending on details of the test and should be chosen so as to “preserve” as much of the structure as possible. For the first option, the only preserved quantity is the density of the network.

The first model—the Erdős–Rényi random graph—has been used in economics (see, for example, [42] for industrial agglomeration, [43] for international trade, as well as [36, 44–46] for the analysis of core-periphery structure). All trade interactions are equiprobable independently of the nodes’ in- and out-degree. In the randomization procedure, the original links are re-assigned to randomly selected pairs of nodes with the probability that is equal to network density *m*/(*n*^2^−*n*).

The next model is the so-called row/column proportional model. The probability that there is an export flow from country *i* to country *j* is proportional to both out-degree of *i* and in-degree of *j*. Examples of null models that belong to this family include Chung-Lu model [47] and the maximum-entropy model [48, 49]. The maximum-entropy model assumes a system of 2*n* equations

$$\begin{array}{ccc} d_{i}^{in} & = & \sum_{j\neq i}\frac{x_{i}^{in}x_{j}^{out}}{1+x_{i}^{in}x_{j}^{out}} \end{array}$$

$$\begin{array}{ccc} d_{i}^{out} & = & \sum_{j\neq i}\frac{x_{i}^{out}x_{j}^{in}}{1+x_{i}^{out}x_{j}^{in}} \end{array}$$

for finding the hidden variables $x_{i}^{out},x_{i}^{in}$. A random graph (null model) is produced with *n* vertices and the probability of forming a directed link *i* → *j* between vertices *i* and *j* is given by

$$\begin{array}{c} p_{ij}=\frac{x_{i}^{out}x_{j}^{in}}{1+x_{i}^{out}x_{j}^{in}}. \end{array}$$

The expected in- and out-degree of the null model is equal to observed in- and out-degrees of the original graph.

The third class of null models assumes that rows/columns are fixed. The in- and out-degree for each vertex is exactly preserved. These models are also known as configuration models. We now describe a simple algorithm that preserves the observed sequence $d_{1}^{out},\ldots ,d_{n}^{out}$. The algorithm has two steps: first, choose two rows arbitrary: *i*^⋆^ and *i*^⋆⋆^. The two rows are compared, in order to identify the set of countries present in *i*^⋆^ but not in *i*^⋆⋆^ and the set of countries present in *i*^⋆⋆^ but not in *i*^⋆^. Second, a certain number of countries that are presented only in the row *i*^⋆^ (but not in the row *i*^⋆⋆^) are replaced with an equal number of countries that are presented only in the row *i*^⋆⋆^ (and not in the row *i*^⋆^). The two steps are reiterated for a certain number of times, resulting in a randomized adjacency matrix without altering the sequence $d_{1}^{out},\ldots ,d_{n}^{out}$. The same procedure is applied for the sequence $d_{1}^{in},\ldots ,d_{n}^{in}$.

#### Core-periphery characteristics

Assume *G* contains a non-empty (*k*_1_, *l*_1_)-core. In this case, the vertex set *V* is a union of two nonempty disjoint subsets: core *V*^*c*^ = *V*_1_ and periphery *V*^*p*^ = *V*\*V*_1_. The graph *G* can be represented as a union of three induced sub-graphs, one of which is bipartite digraph, *G*^*cp*^, that is:

$$\begin{array}{c} G=G^{c}\cup G^{p}\cup G^{cp} \end{array}$$

Let *n*_*c*_ = |*V*^*c*^| and *n*_*p*_ = |*V*^*p*^| be the number of vertices in the core and the periphery, respectively. For a given core-periphery structure we compute density *ρ* and the trade flow *S* as follows:

$$\begin{array}{ccc} \rho^{\alpha } & = & \frac{m_{\alpha }}{n_{\alpha }\left(n_{\alpha }-1\right)} \end{array}$$ (3)

$$\begin{array}{ccc} \rho^{\alpha \beta } & = & \frac{m_{\alpha \beta }}{n_{\alpha }n_{\beta }} \end{array}$$ (4)

$$\begin{array}{ccc} S^{\alpha \beta } & = & \frac{\sum_{i\in V^{\alpha }}\sum_{j\in V^{\beta }}w_{ij}}{\sum_{i}\sum_{j}w_{ij}} \end{array}$$ (5)

where *α*, *β* ∈ {*c*, *p*} (*c* for core and *p* for periphery), *m*_*α*_ is the number of links in *α*, and *m*_*αβ*_ is the number of links from *α* to *β*.

## Results and discussion

### Block reliability and refinement vis-à-vis previous research

In this section we will first briefly review the results of LARDEG core-periphery model. Next, we will compare results obtained from our algorithm to those from alternative models, that is (1) the deterministic block model for weighted directed networks that uses regular equivalence implemented with REGE algorithm; (2) the SBM for weighted directed networks; (3) the continuous multiplicative core-periphery model for weighted directed networks, implemented with MINRES/SVD algorithm. The first model is the only one used so far for the analysis of sectoral international trade networks. SBMs are popular models for learning mesoscale structures (especially community structures) in networks. The third model is the only one for computing “in-coreness” and “out-coreness” in weighted directed network, although does not recover the core-periphery structure. Moreover, it has also been used for the analysis of trade networks.

**Fig 1:**
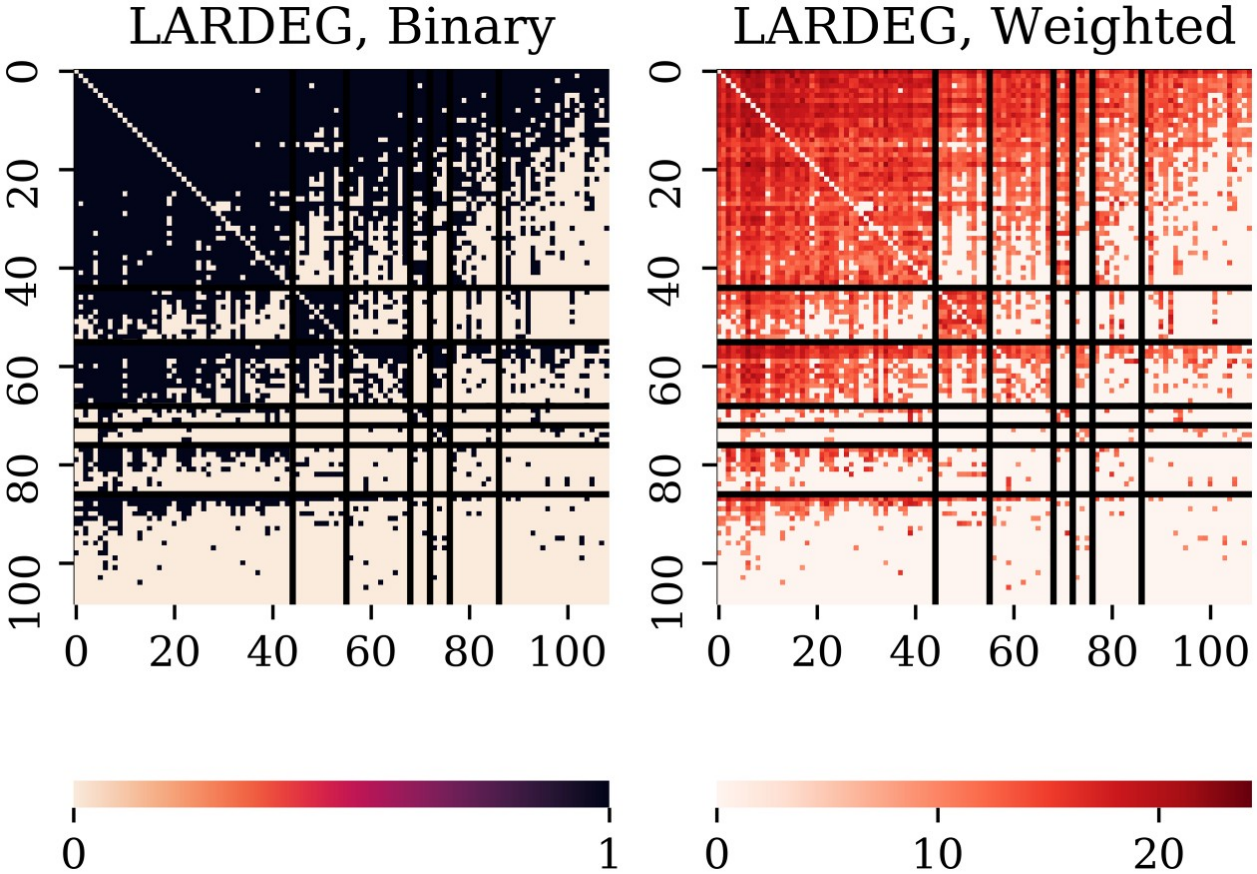
Adjacency matrix of the sector 84 (Articles of apparel and clothing accessories) and the year 2016. The vertex set of the binary network is partitioned with the LARDEG model. Left panel: binary adjacency matrix. Right panel: same as the left panel colored with weighs. In both panels, the horizontal and vertical lines indicate the subsets of the vertex set.

The LARDEG model partitions the vertex set into *r* disjoint subsets (groups, blocks)

$$\begin{array}{c} V=V_{1}\cup V_{2}\ldots \cup V_{r} \end{array}$$ (6)

such that, within the group (block) *V*_*i*_, each member of the group has at least *k*_*i*_ incoming links to other members of *V*_*i*_ and at least *l*_*i*_ outgoing links from other members of *V*_*i*_. Since *V*_*i*_ is the set for which *k*_*i*_ and *l*_*i*_ are maximal, it contains countries with largest export/import collaboration among members of the same group *V*_*i*_. Hence, by implementing the procedure discussed above, the vertex set of each network is partitioned into *r* blocks, with *r* ranging from 4 to 9 (depending on the sector). Basically, our mechanism produces networks with nested core-periphery structure, or blocks (groups) existing at several levels. The next subsection provides the evidence about the reasons for country groupings at first, second, and third hierarchical level. Fig 1 shows an illustrative substantive application (example) of our proposed model to international trade in a selected SITC category (84 Articles of apparel and clothing accessories) for the year 2016. Moreover, it yields clear and reasonably robust interpretation concerning the overall structure of both the binary and weighted directed networks. For this network (sector 84), *r* = 7 and |*V*_*i*_| and (*k*_*i*_, *l*_*i*_) are found to be

$$\begin{array}{ccc} & & \begin{array}{ccccc} i= & 1 & 2 & 3 & 4\\ |V_{i}|= & 45 & 11 & 13 & 4\\ (k_{i},l_{i})= & \left(37,38\right) & \left(8,8\right) & \left(5,5\right) & \left(2,2\right) \end{array} \end{array}$$

$$\begin{array}{c} \begin{array}{cccc} i= & 5 & 6 & 7\\ |V_{i}|= & 4 & 10 & 22\\ (k_{i},l_{i})= & \left(2,1\right) & \left(1,1\right) & \left(0,0\right) \end{array} \end{array}$$ (7)

In view of space constraints here, the partitioning of all other vertex sets and information thereof is provided in S1 File.

Eq (6) describes the nested block structure such that each pair (*V*_*i*_, *U*_*i*_) where $U_{i}=\cup_{k=i+1}^{r}V_{k})$, for *i* = 1, …, *r*−1, is called *i*-level structure. The first-level block defines the core-periphery structure specified as follows: the core *V*^*c*^ = *V*_1_ and the periphery *V*^*p*^ = *V*\*V*_1_. For each trade network and the first-level core-periphery structure, we compute densities and trade flows (see Eqs (3), (4) and (5)). Thus, for example, for the sector 84 and the year 2016 (see Table 1), we found that the density (*ρ*) for the whole network is *ρ* = 0.454, while the densities and trade flows (in percentage) of the core-periphery structure are given as follows:

$$\begin{array}{c} \rho^{c}=0.962,\rho^{p}=0.171,\rho^{cp}=0.581,\rho^{pc}=0.374\\ S^{c}=79.95\%,S^{p}=0.69\%,S^{cp}=8.35\%,S^{pc}=11.01\% \end{array}$$

**Table 1:** Densities and trade flows at the first hierarchical level (selected SITC industries, 2016).

Sec	*ρ*	*ρ*^*c*^	*ρ*^*p*^	*ρ*^*cp*^	*ρ*^*pc*^	*S*^*c*^	*S*^*p*^	*S*^*cp*^	*S*^*pc*^
**01**	0.239	0.83	0.122	0.3933	0.1476	0.5054	0.1587	0.2334	0.1026
**06**	0.328	0.814	0.113	0.4052	0.2402	0.6592	0.0433	0.1771	0.1204
**07**	0.412	0.918	0.137	0.5642	0.4139	0.7054	0.0204	0.1243	0.1499
**12**	0.229	0.794	0.08	0.3567	0.2281	0.5442	0.0834	0.2241	0.1483
**28**	0.241	0.828	0.065	0.2368	0.3995	0.6376	0.0232	0.055	0.2843
**33**	0.331	0.877	0.116	0.5567	0.2157	0.6401	0.0301	0.1951	0.1347
**34**	0.092	0.894	0.054	0.2713	0.1073	0.3233	0.3226	0.1507	0.2034
**41**	0.136	0.708	0.045	0.279	0.1085	0.6481	0.045	0.187	0.1198
**54**	0.431	0.929	0.141	0.6609	0.1817	0.9324	0.0074	0.0576	0.0026
**71**	0.435	0.956	0.125	0.6646	0.2519	0.8899	0.0024	0.0964	0.0113
**72**	0.521	0.987	0.2	0.7735	0.3387	0.867	0.006	0.118	0.009
**73**	0.331	0.944	0.076	0.5423	0.1768	0.8964	0.002	0.093	0.0086
**78**	0.494	0.98	0.161	0.7557	0.3326	0.9069	0.0025	0.078	0.0126
**84**	0.454	0.962	0.171	0.5806	0.3743	0.7995	0.0069	0.0835	0.1101

Data for the density of the networks, *ρ*, the densities for the first core (c)—periphery (p) structure: *ρ*^*c*^, *ρ*^*p*^, *ρ*^*cp*^, *ρ*^*pc*^ and the trade flows within and between core (c) and periphery (p) blocks *S*^*c*^, *S*^*p*^, *S*^*cp*^, and *S*^*pc*^.

**Fig 2:**
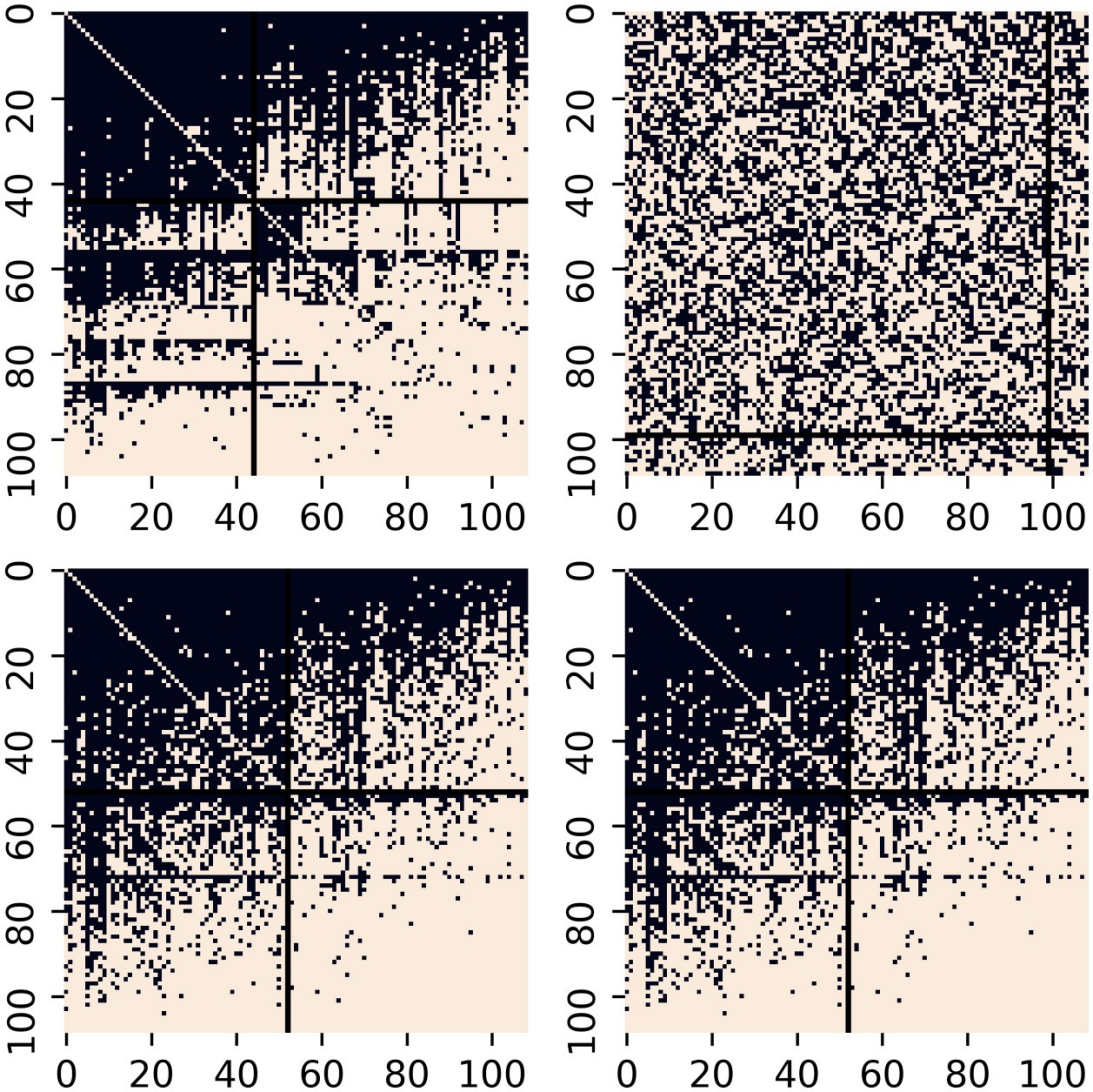
Binary adjacency matrices of sector 84 (Articles of apparel and clothing accessories) and year 2016. Partitioned with LARDEG model (upper left panel) and three null models: preserving the density of the network (upper right panel), preserving, on average, in- and out-degree of the nodes (lower left panel), and preserving exactly in- and out-degree of the nodes (lower right panel). In all four panels, the horizontal and vertical lines indicate the the first subset of the vertex set obtained with the LARDEG model. The numbers represent the number of vertices in the first subset for each model.

Borgatti and Everett [36] define an ideal core-periphery structure with *ρ*^*c*^ = 1, *ρ*^*p*^ = 0, *ρ*^*cp*^ = 1, and *ρ*^*pc*^ = 1. However, these ideal structures are rarely found in real networks. Since *ρ*^*c*^ ≫ *ρ*^*p*^ and *ρ*^*pc*^, *ρ*^*cp*^ > *ρ*^*p*^ for all networks analyzed here, we may well conclude that our first-level structure is indeed a core-periphery. Moreover, as mentioned previously, we consider three null models to examine the first-level core-periphery structure in trade networks. For example, Fig 2 shows adjacency matrices obtained for the null models related to sector 84 and year 2016. When analyzing core-periphery structures in trade networks, null models aim to address the question on whether the observed core-periphery structure may be reproduced by some simple random mechanisms of network generation. The figure indicates that the density of the network does not reproduce to any extent the core-periphery structure of the network. The second and the third null models (lower left and right panels) reproduce only the first level/layer core-periphery structure.

**Fig 3:**
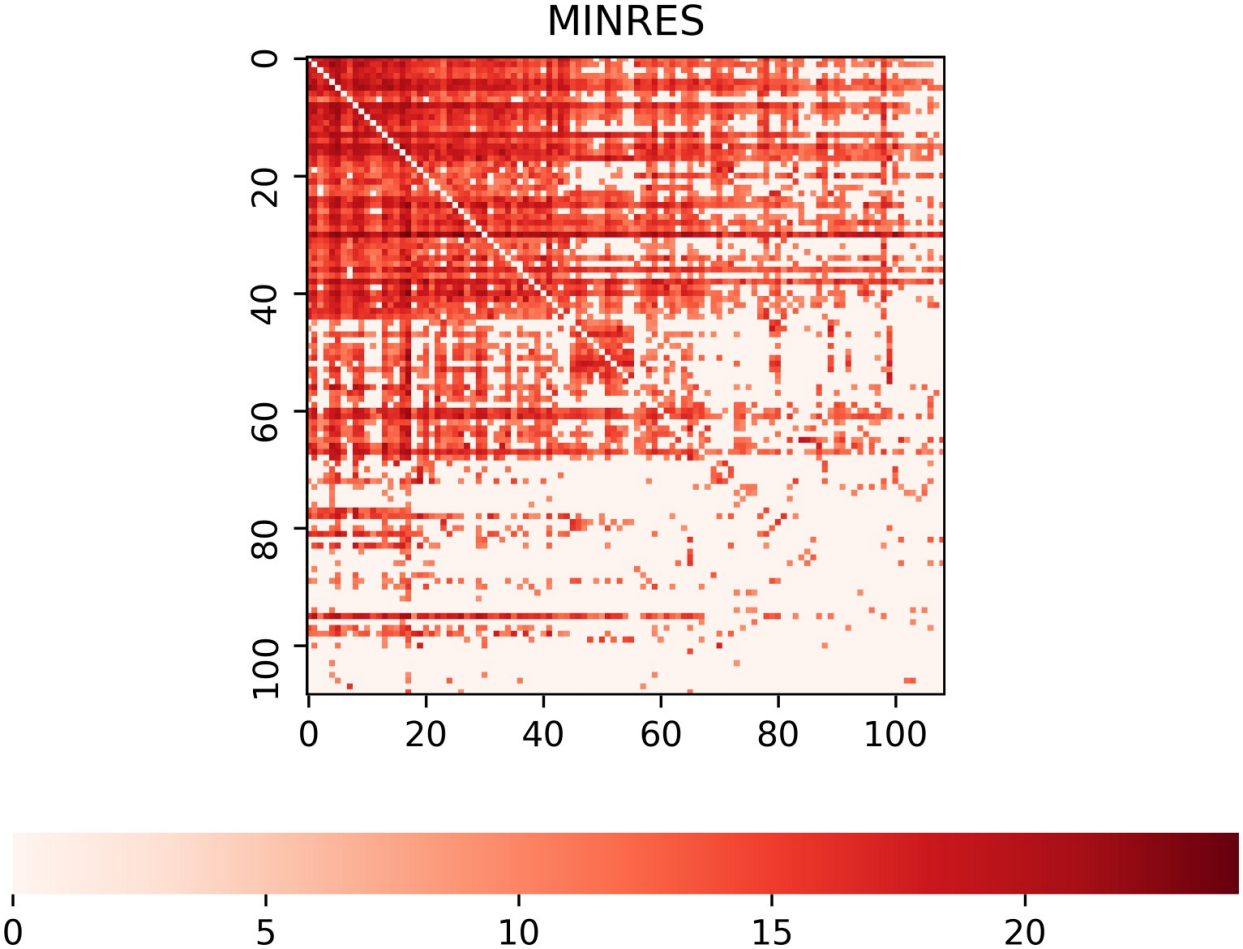
Weighted adjacency matrix of the network 84 (Articles of apparel and clothing accessories) and the year 2016. Counties are ordered according to “in-coreness” and then according to “out-coreness” computed with the MINRES/SVD algorithm.

Next, we will compare results of our algorithm for the selected industry (84) to those obtained from alternative models. First, we compute the quantities “in-coreness” and “out-coreness” using the MINRES/SVD algorithm. The algorithm does not partition the vertex set into blocks. However, if we compare Fig 1 (right panel) and Fig 3, the core-periphery structure of trade network becomes clearly visible.

**Fig 4:**
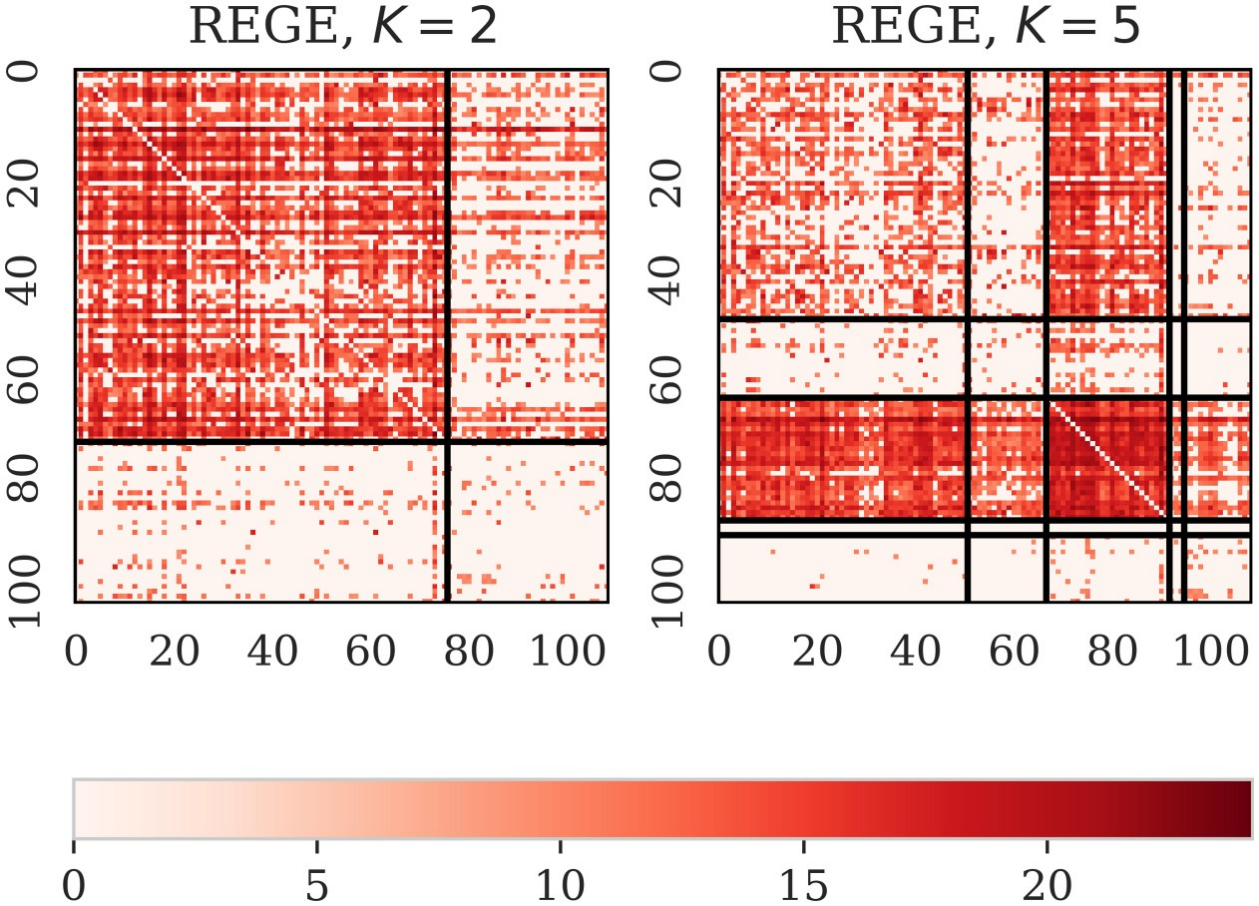
Adjacency matrices of the sector 84 (Articles of apparel and clothing accessories) and the year 2016. Partitioning with REGE: model with two blocks (left panel) and model with five blocks (right panel).

**Fig 5:**
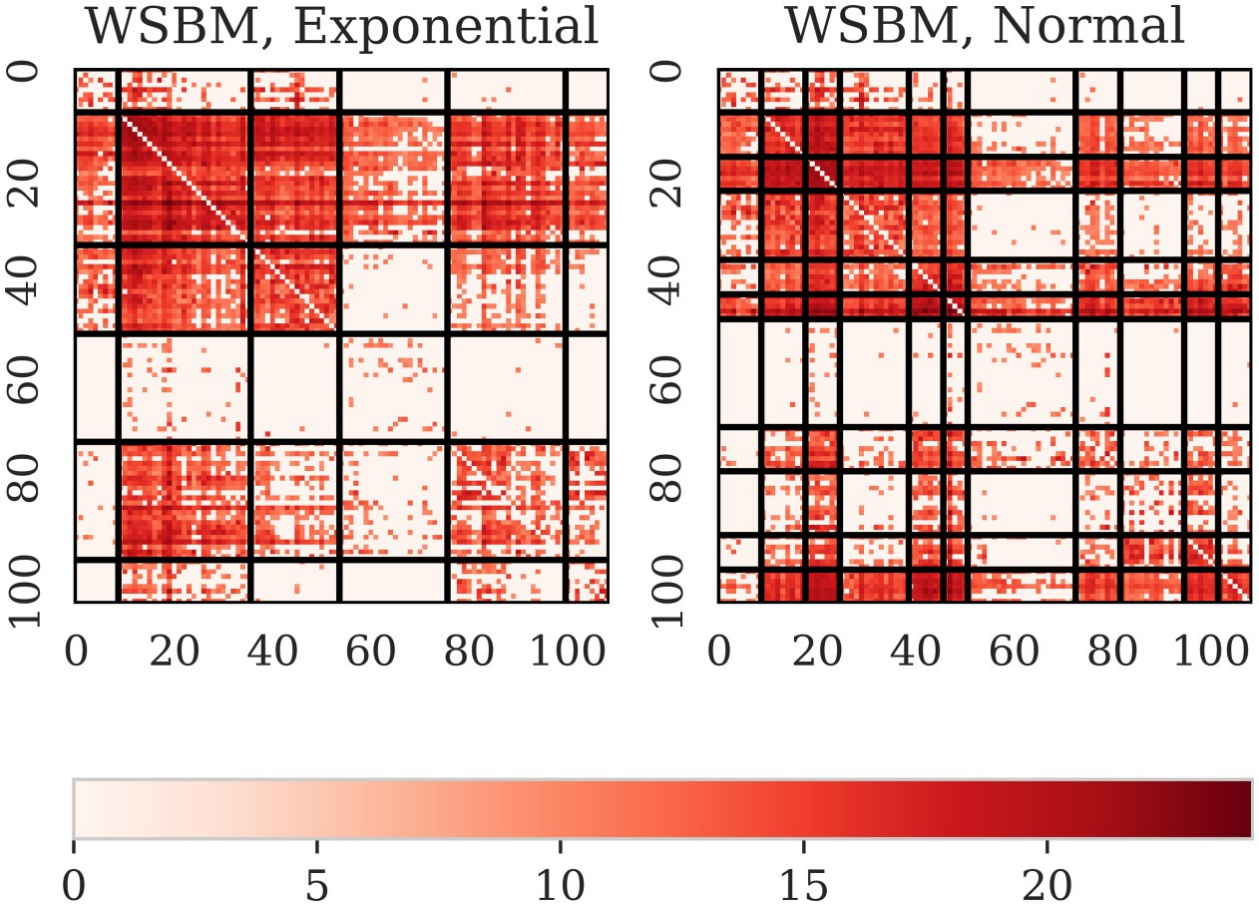
Adjacency matrices of the sector 84 (Articles of apparel and clothing accessories) and the year 2016. Partitioning with the weighted stochastic block model: exponential model (left panel) and normal model (right panel).

Second, we compare the groups obtained with LARDEG model to those produced by (1) block modeling based on regular equivalence and (2) the stochastic block modeling. Figs 4 and 5 summarize our findings. The output of REGE class of algorithms, (see [50]), is a set of hierarchically nested blocks such that succeeding partitions break up the blocks of the previous partition into smaller blocks whose members are to some extent more equivalent. Fig 4 shows two such partitions with two and five blocks (left and right panels, respectively). REGE algorithm is, however, accompanied with several limitations: it runs in time proportional to *n*^5^, lacks a theoretical rationale for the produced similarity measure, and requires a priori knowledge of the number of groups (clusters). Fig 5 depicts the partitioning of trade network for the sector 84 and the year 2016 using weighted stochastic block models developed recently by Peixoto [31]. We consider two models for the weights: (1) exponential model applied to the original data (left panel), and (2) normal model applied to the transformed data resulting in a log-normal model for the weights (right panel). Networks, however, exhibit far more complex structure than can be captured by current SBMs (see [51]). The LARDEG model, in contrast, (1) runs in time proportional to the number of edges (and, hence, is optimal), (2) is a parameter-free, (3) is supported with a graph-theoretical rationale based on representing collaborative features of the nodes using their robustness under degeneracy, and (4) has clear economic grounds both in in-degree and out-degree, i.e. import and export relationships between countries are tightly connected to their GDPs.

As already mentioned, SITC category 84 is taken for illustration and comparison purposes only. However, our model is used to make partitions of all networks into blocks (6). In this context, several key observations can be drawn from the analysis conducted here:

- The first block *V*_1_ contains those countries in the network that have largest export/import collaboration among the first-level block members. Moreover, the pair (*V*_1_, *V*\*V*_1_) describes a real core-periphery structure. In the five strata terminoolgy suggested by Smith and White [21], this block could be called a “core”.
- The last block *V*_*r*_ contains those countries for which *k*_*r*_ = 0 and *l*_*r*_ = 0. This block includes isolated countries or almost isolated countries (that is, countries with only one out-going link or one in-coming link) with respect to other countries in the same block. In Smith and White’s [21] five strata terminology, this block could correspond to a “weak periphery”.
- The blocks *V*_2_, …, *V*_*r*−1_ have different structures yet to be investigated. Some of the blocks could be grouped together forming larger clusters. Whether and how some of these blocks/clusters could be related to other three structures suggested in Smith and White [21], namely, “strong semi-periphery, weak semi-periphery, and strong periphery”, is an open question.

### Country groupings at different hierarchical levels

Our analysis observes the core block of a network as a core of the market in a particular industry—the sub-group of countries that, having a great number of edges, tend to accept the role of market-makers. Tables 2 and 3 report the number of countries belonging to the first-level core blocks of selected SITC industries at different points in time (detailed information on all sectors/years is provided in S1 File). Obviously, the core block is made up of tightly interconnected exporters and importers. The subgraphs formed by these groups of strongly linked countries show a density that has been almost consistently higher than 0.70 (see Tables 2 and 3; detailed information on all sectors/years is provided in S1 File). In an attempt to analyze the reasons behind the structure of country groupings at this level of sectoral international trade networks, we look first at the economic dimension of countries, whose proxy variable here is total GDP (current US$). GDP represents the commonly designated ‘size effect’, i.e. a phenomenon where larger countries trade more than smaller ones [18, 52]. The evidence suggests that the core countries have the largest markets all over the world. The USA, China, Japan, Germany, United Kingdom, France and Italy are the most interconnected countries in the world appearing in core groups of more than 50 industries for all points in time (see Table 4 for selected countries/years; detailed information on all countries/years is provided in S2 File).

**Table 2:** Basic data for the first-, second-, third-level core (selected SITC industries, 2000).

Sec	*n*_1_	*k*_1_	*l*_1_	*ρ*_1_	*n*_2_	*k*_2_	*l*_2_	*ρ*_2_	*n*_3_	*k*_3_	*l*_3_	*ρ*_3_
**04**	33	20	20	0.861	10	6	6	0.8667	10	4	4	0.6889
**09**	27	23	23	0.967	10	5	7	0.8778	11	5	5	0.7636
**29**	37	25	23	0.883	11	6	6	0.7909	7	4	3	0.7619
**54**	36	29	28	0.956	11	6	6	0.8182	10	5	5	0.8
**55**	39	29	24	0.893	11	5	4	0.7273	12	4	4	0.6061
**65**	43	37	36	0.972	11	8	8	0.9182	11	6	5	0.7727
**72**	39	34	33	0.981	12	5	5	0.697	10	5	4	0.6778

Number of nodes *n*_*i*_, (*k*_*i*_, *l*_*i*_), and the density *ρ*_*i*_ for the first three cores.

**Table 3:** Basic data for the first-, second-, third-level core (selected SITC industries, 2016).

Sec	*n*_1_	*k*_1_	*l*_1_	*ρ*_1_	*n*_2_	*k*_2_	*l*_2_	*ρ*_2_	*n*_3_	*k*_3_	*l*_3_	*ρ*_3_
**02**	26	19	18	0.928	20	10	9	0.7763	11	4	4	0.6
**22**	21	15	17	0.943	12	6	4	0.697	9	4	4	0.7361
**54**	48	36	33	0.929	10	7	7	0.9444	8	5	5	0.8929
**69**	52	45	43	0.976	10	8	8	0.9778	13	4	4	0.5641
**74**	51	44	44	0.985	13	8	6	0.7756	14	6	5	0.6099
**77**	51	45	45	0.988	14	8	7	0.8077	11	5	4	0.6727
**84**	45	37	38	0.962	11	8	8	0.9364	13	5	5	0.6603

Number of nodes *n*_*i*_, (*k*_*i*_, *l*_*i*_), and the density *ρ*_*i*_ for the first three cores.

**Table 4:** Number of industries the country appears at the first-level core blocks.

Country Code	Industries 2000	Country Code	Industries 2009	Country Code	Industries 2016
DEU	63	ITA	63	ITA	63
NLD	63	DEU	63	DEU	63
BEL	62	NLD	62	GBR	62
GBR	62	BEL	62	NLD	62
FRA	62	GBR	62	ESP	62
ITA	61	USA	62	USA	62
USA	61	FRA	61	BEL	62
ESP	60	ESP	60	FRA	62
CAN	59	POL	58	POL	61
CHN	58	CHN	57	CZE	59
SWE	56	SWE	57	CHN	58
DNK	55	DNK	57	AUT	57
AUS	54	CHE	56	CAN	57
CHE	53	CAN	55	HUN	56
JPN	53	AUT	55	CHE	56
AUT	51	IND	54	SWE	55
MYS	50	CZE	54	DNK	55
IND	49	JPN	51	ROU	54
ZAF	49	TUR	51	RUS	53
SGP	47	AUS	51	GRC	53
KOR	47	HUN	49	IND	52
THA	47	THA	48	TUR	52
HKG	46	MYS	48	KOR	51
HUN	46	KOR	48	JPN	51

The position of Canada and Mexico is also worth mentioning. The North American Free Trade Agreement (NAFTA) has made the two countries closely linked to the USA for more than 20 years. Although the various phases of economic cycles toss economies around the world, the largest economic countries remain nevertheless the same. When compared to the top 20 economies of 2000, 18 are still present on the list which means only two new entrants in 2016. These countries are also the engine of growth, dominating majority of the global wealth. The nominal GDP of the top 10 economies adds up to about 71% of the world economy in 2016, while the top 20 economies contribute almost 85%. Thus, they tend to have large exports and imports to and from many countries dispersed all over the world economy. World-system analysts predict that core status should be correlated with higher scores of GNI per capita (the so-called ‘income effect’) or other indicators for the level of development. The average levels of GNI per capita reinforce the view about the high-income status (i.e. GNI per capita of $12,376 or more according to the World Bank country classification into income groupings) for all core blocks of the various sectoral international trade networks (data on all sectors/years is provided in S3 File). However, closer analysis of differences between the individual levels of GNI per capita figures suggests that this indicator is a much poorer proxy for the world-system status. Oman, for example, fits clearly into our periphery structure at different time points. Like other petroleum-producing countries (e.g. Bahrain, Kuwait etc.), it lacks a diversified industrial economy but has a GNI per capita corresponding to that of the high-income core economies. India, on the other hand, has a GNI per capita either at parity with the poorest countries in the world (e.g. Tanzania and Yemen in 2000), or similar to the lower middle-income economies in 2016. However, the country’s diversified industrial production split among various manufacturing sub-sectors, the position of being one of the largest start-up hubs in the world, as well as the growing integration into the global economy explain why India fits into the core group of 49 and 52 industries in 2000 and 2016, respectively (see Table 4).

The apparent reduction in density and numbers behind the two positive integers (*k* and *l*) is particularly noticeable in blocks we label as second- or third-level ‘core’ (see Tables 2 and 3 for selected SITC industries at different points in time; detailed information on all sectors/years is provided in S1 File). Given that these subgroups arise from the iterative decomposition of the network periphery, we have arbitrarily decided to analyze the membership of those blocks performing ‘reasonably well’ in both the density and number of nodes (e.g. the lowest density score of 0.65 and minimum block size of 10 nodes for the second-level groups). Taken together, the evidence implies that, unlike the first level, patterns of ‘cores’ here are strongly dependent on distance and geographical proximity. The analysis finds interesting the case of some countries previously holding a peripheral position but maintaining preferential ties at second layer of the sectoral international trade networks (e.g. Northern, Eastern and Southern European countries in 2000, as well as the countries from Central and South America both in 2009 and 2016). Intra-regional trade is also high among the group of Eastern and South-East Asian countries at all points in time. Put differently, international trade for many groups at this level is highly regionalized and/or structured around the trading blocs, possibly created by regional agreements (see for example [53, 54]). The regional trade agreements seem to reinforce the ‘proximity effect” for the set of South-East Asian countries belonging to ASEAN Free Trade Area (AFTA) and the South American constituents of MERCOSUR. Similarly, a decade before joining the European Union in 2004 and 2007, the CEFTA membership had benefited most of the Central and Eastern European countries by freeing trade [55]. The existence of regional trade agreements supporting trade between similar and often neighboring countries can explain the higher number of strong trade links for all members in the region.

As we move across the third layer of the sectoral international trade networks, the density of the blocks reduces even further. Looking at the most representative country groupings in terms of size and density, one may notice a substantial share of regional trade existing at this level as well. The most prominent positions are held by the major oil exporting countries of Middle East, as well as the countries from Northern Africa (Morocco, Tunisia and Algeria) and West Africa (e.g. Benin, Burkina Faso, Cote d’Ivoire, Gambia, Niger, Senegal and Togo). There is obviously much more political enthusiasm for regional integration instead of unilateral trade liberalization. Most African countries belong to a number of regional trade agreements set to promote trade among themselves (e.g. West African Economic and Monetary Union as one of the most functioning customs union in Africa). It is worth noting that Africa accounts for a small share of the world trade and the central position held by African countries at this level is also attributed to its strong dependence on (i.e. the attracting force of) European (especially for the close neighbors of North Africa) and Asian major players, as well as the lack of viable alternatives.

### Relations between blocks: Trade asymmetries and mobility

The structural theories of world-economy reinforce the dominance of an intra-core trade and a great likelihood of the peripherals to trade with the core nations that they have been historically dependent upon than they are to trade with the other peripheral countries [4]. World-system theorists propose asymmetrical flows of goods resulting from an unequal trade between the peripheral-produced simple (resource and labor exploiting) products and the knowledge-intensive (value-added products) from the core countries [4]. While seventeen years is not a long period for macrostructural transformation in the patterns of global trade, these asymmetries do provide important information about the implications of unbalanced trade flows for global inequality and offer empirical evidence for recent major changes to the world economy (e.g. the growth of global value chains (GVCs) that have reshaped the international division of labor).

The results of our analysis allow us to look at 630 sectoral international trade networks. Clearly, detailed analysis of such a large amount of data must wait for another paper. Instead, two valuable attributes will be examined here. One is the percentage of the total trade value that is exchanged both within (among the core countries) and between blocks (core and periphery) at the first hierarchical level. The other looks exclusively at the patterns of country mobility between blocks to estimate the positive and negative effects of both globalization and the rise of global value chains (GVCs). Tables 1 and 5 contain the percentage of intra-core and inter-block trade for selected industries at two time points (the results for all industries/years are provided in S4 File).

**Table 5:** Densities and trade flows at the first hierarchical level (selected SITC industries, 2000).

Sec	*ρ*	*ρ*^*c*^	*ρ*^*p*^	*ρ*^*cp*^	*ρ*^*pc*^	*S*^*c*^	*S*^*p*^	*S*^*cp*^	*S*^*pc*^
**01**	0.188	0.897	0.073	0.5275	0.2034	0.5238	0.0418	0.3669	0.0676
**06**	0.237	0.8	0.094	0.36	0.2014	0.5545	0.1569	0.1559	0.1327
**07**	0.305	0.829	0.1	0.4177	0.2973	0.6614	0.0317	0.1052	0.2017
**12**	0.188	0.778	0.065	0.3767	0.1816	0.5483	0.0501	0.3233	0.0784
**28**	0.195	0.804	0.05	0.2018	0.3143	0.6894	0.0251	0.0567	0.2287
**33**	0.246	0.729	0.07	0.3685	0.1707	0.6858	0.0355	0.0736	0.2052
**34**	0.063	0.945	0.032	0.234	0.0561	0.2352	0.2984	0.0589	0.4075
**41**	0.102	0.8	0.035	0.311	0.1099	0.4136	0.0877	0.344	0.1546
**54**	0.343	0.956	0.119	0.6457	0.1937	0.8854	0.0134	0.0951	0.0062
**71**	0.329	0.956	0.077	0.6002	0.2286	0.9268	0.0012	0.0609	0.011
**72**	0.394	0.981	0.109	0.6857	0.2883	0.9001	0.0023	0.0875	0.0101
**73**	0.256	0.906	0.043	0.4388	0.1294	0.9064	0.0016	0.0829	0.0092
**78**	0.387	0.972	0.118	0.7012	0.2954	0.9266	0.0016	0.0588	0.0131
**84**	0.364	0.882	0.082	0.4879	0.2505	0.8969	0.003	0.0504	0.0496

Data for the density of the networks, *ρ*, the densities for the first core (c)—periphery (p) structure: *ρ*^*c*^, *ρ*^*p*^, *ρ*^*cp*^, *ρ*^*pc*^ and the trade flows within and between core (c) and periphery (p) blocks *S*^*c*^, *S*^*p*^, *S*^*cp*^, and *S*^*pc*^.

The evidence suggests that values of intra-core trade for vast majority of manufactures (SITC 5-8) are much higher (80% and above) than those recorded for primary products (SITC 0-4). For example, intra-core trade predominates (around or above 90%) for leading manufacturing industries such as pharmaceuticals (54) and machinery and transport equipment (71, 72, 73, 78). The pattern is also entirely consistent with the theoretical expectations that high-technology manufactures are likely to be exported from the core to periphery. Indeed, the core-periphery trade flows are relatively stable for pharmaceuticals (54), while they are on the rise for the ‘power generating machinery and equipment’ (71), ‘machinery specialized for particular industries’ (72), ‘metal working machinery’ (73) and ‘road vehicles’ (78). At the same time, the flows coming from the periphery to core structures of these industries consistently show negligible results (trade share of 1% or even less). However, closer analysis of manufactured goods reveals that the structure of ‘apparel and clothing’ network (84) changed dramatically from 2000 to 2016 (see Tables 1 and 5). The sector shows a significant decrease in the share of intra-core trade (90% and 80% in 2000 and 2016, respectively) at the expanse of flows coming from the periphery toward consumption in the core (5% and 11% in 2000 and 2016, respectively). In point of fact, the decrease in relative importance of North America and Western Europe in global textile and apparel production was offset by the increase in global value added shares of Eastern Asia, South-Central and South-East Asia and South America. The labor intensity and requirements for huge number of unskilled workers has made textile and clothing a widely recognized sector providing good opportunities for industrialization. Production moves quickly between countries in search for low-cost labor. As yet, however, there is little sign that most of developing countries will be able to enjoy the resounding success of China, Hong Kong and Korea. Entrepreneurs from developing countries who want to mimic the East Asian strategic suppliers will have to upgrade the necessary skills and capacity (including in ICT), as well as to achieve greater speed and flexibility in reaching markets (see [56] and [9]) for discussions about the most important mechanisms facilitating the shift to higher value-added activities in apparel industry, e.g. the triangle production networks established by East Asian suppliers).

Further examining the results, we find that sectors of food (SITC 0) and raw materials (SITC 2 and 4) and energy products (SITC 3), unlike manufactures, get much higher scores for trade flows moving from periphery to core blocks. The extractive industries (oil, gas and mining) are the most obvious example for low-level processing commodities supporting such movements (see Tables 1 and 5 for the years 2016 and 2000; the results for all years are provided in S4 File). Metalliferous ore and metal scarp (28), for instance, shows an increased share of trade value that our periphery [e.g. West Africa (Mauritania, Burkina Faso), Southern Africa (Botswana, Namibia), Eastern Africa (Mozambique, Tanzania, Zimbabwe), Central African Republic, Western Asia (Lebanon, Armenia)] has exchanged with the countries of the core block (23% and 28% in 2000 and 2016, respectively). The patterns for the remaining two groups of extractive products are more varied and prone to higher fluctuations over time. For example, the gas, natural and manufactured (34) has recorded a sharp reduce in the share of periphery-core trade value ranging from 41% in 2000 to 20% in 2016. There is also a gradual but continuous decline in the share of the comparable trade movements for petroleum and petroleum products (33). These unstable patterns may be of intrinsic interest, since they are indicative of the end of the commodity ‘super cycle’ that characterized the early 2000s, and rather suggestive of both the substantial shifts in OPEC’s policy objectives and the spillovers from geopolitical risks [57] [58]. The standard argument about trade asymmetry between different blocks over the years (i.e. high technology production is centered in the core and commodities at low levels of processing originate from lower levels of the world-economy) seems to apply to agricultural sectors as well (see for example the percentage share of periphery-core trade flows for ‘meat and meat preparations’ (01), ‘sugars’ (06), ‘coffee, tea, cocoa and spices’ (07), ‘tobacco and tobacco manufactures’ (12), ‘animal oils and fats’ (41), etc.). However, it is even more noteworthy that large majority of food production in our core blocks is destined for markets in other core states. Industrial development involves, among others, shifting the production factors from traditional to modern agriculture. Nevertheless, despite the importance of this sector for developing countries and their comparative advantage in agricultural production, participation of developing countries in GVCs appears to be more limited than in other sectors.

Overall, the differences in levels of processing between the higher and lower blocks of the core/periphery hierarchy, and unequal patterns of change therein, shows that the rise of global production networks is far from an equal diffusion of technology. The pattern of country mobility between different blocks (at first hierarchical level) is another attribute on which to measure the alleged change via global production networks and the ensuing world division of labor. The empirical evidence suggests that there is a high level of continuity in the system as a whole. This is obvious when looking at the top twelve countries of the hierarchy appearing in core blocks of more than fifty industries at all times (see Table 4). Basically, the structure of the networks is both core-periphery and multipolar, with a leading role played by the major European countries (e.g. Germany, Netherlands, Belgium, United Kingdom, France, Italy, Spain) and United States, as well as the emerging market of China acting as a third pole. Even the financial crisis of 2008 did not change the core positions in 2009 (see Table 4). This would also imply that the structure of the GVC networks expressed by the topology of country to country relationships is resilient, even when the economic shocks of great size hit the global economy (see [59] for recent patterns of global production and GVC participation). It is also noteworthy that, irrespective of the year, neither a Central American nor an African country (except South Africa, which is the most industrialized and diversified economy on the continent of Africa, and acts as a regional manufacturing hub), appears among the major world players in almost all sectors.

Movement between blocks is another type of possible change that our methodological tools allow to examine, by constructing inter-block mobility tables across the years (see Table 6) for selected industries/years; data on all industries/years is provided in the S5 File). Debates about the nature of the rise and fall of nations flourish in international political economy. We make no pretense here to provide a comprehensive description of our empirical results in the brief discussion that follows. Instead, we would suggest that mobility between our blocks may be related to the rise of global value chains and the consequent changes in the world division of labor over the past two decades. Clearly, the analysis finds much more upward than downward mobility in international system, with Viet Nam, Mexico, United Arab Emirates, and the countries of Central and Eastern Europe (Bulgaria, Romania, Slovakia, Poland, Croatia, Slovenia, Estonia, Latvia, Lithuania) making one of the greatest jumps between 2000 and 2016 (for selected industries, see Table 6). The successful diversification of industrial production and trade patterns can actually explain why the Emirates (as the second largest economy in the Middle East) fits into our core blocks of 22 and 37 industries in 2000 and 2016, respectively. Strong production links between Eastern and Western Europe already existed in 1990 and have reinforced further since. The region, for example, has participated in the Western Europe’s production network whilst the value chains of different industries exhibit a strong regional component. Nevertheless, the best development outcome is obtained if both GVC participation and domestic value added content in foreign exports (forward GVC participation) increase at the same time. Unlike some middle-income economies (e.g. Romania) that moved up faster in backward participation (i.e. deepening in assembly and processing as a result of increasing the foreign value-added embedded in gross exports of a country), the forward GVC participation raised more rapidly in high-income countries (e.g. Estonia). Mexico and Viet Nam are examples of economies with high GVC participation growth rates as well. Initially, both countries were recognized as being exporters of primary products, but soon they made a progress toward the manufacturing exports. However, in the absence of opportunities to be involved in higher-level electronics design, Viet Nam is primarily engaged in the production and assembly stage of manufacturing sector (light manufacturing, electrical equipment, electronics etc.) in the GVCs. Mexico’s economic outlook, on the other hand, will likely remain closely linked to that of the U.S, despite the country’s efforts to diversify trade. The importance of one country for the other is not symmetrical, and thus the response to each other policies has varied substantially. There are also some other attributes of national attitude, but historically, the Mexico’s political-economic dependence on the United States, has had a significant impact on bilateral relations between the two neighboring countries.

**Table 6:** Inter-block mobility of countries (new in/left from the first-level core) (selected SITC industries).

Sec	2000-2016 New	2000-2016 Left
**06**	BRA, VNM, RUS, HRV, ROU, EGY, UKR, BGR, SVN, PHL, ARE, SGP	FIN
**11**	CHL, SVK, VNM, LTU, EST, ROU, PRT, UKR, BGR, SVN, TUR, IND, NOR, LVA	MYS, ARG
**54**	SVK, POL, RUS, VNM, LTU, EGY, HRV, ROU, BGR, SVN, LVA, IDN	
**58**	BRA, SVK, POL, VNM, RUS, SAU, EGY, LTU, ROU, UKR, BGR, SVN, ARE, MEX, IDN	
**61**	BRA, SGP, SVK, POL, VNM, HRV, ROU, PAK, HUN, SVN, CZE, MEX, NZL, IDN	JPN, NOR
**64**	VNM, HRV, LTU, EST, PAK, ROU, UKR, BGR, SVN, ARE, SRB, LVA	
**69**	SVK, VNM, HRV, LTU, EST, EGY, PAK, ROU, UKR, BGR, SVN, SRB, LVA	PHL
**71**	SVK, VNM, HRV, LTU, EST, ROU, UKR, BGR, SVN, ISR, ARE, SRB, LVA	MYS, ZAF, IDN, NZL
**75**	GRC, SVK, POL, VNM, RUS, EST, LTU, HRV, ROU, PRT, BGR, SVN, TUR, LVA	NZL, IDN
**76**	SVK, VNM, RUS, LTU, EST, HRV, ROU, BGR, SVN, LVA, IDN	
**77**	VNM, LTU, EST, UKR, ARE, SRB, LVA	
**78**	GRC, SVK, LTU, EST, ROU, BGR, ISR, LVA, NZL	
**83**	BRA, SVK, POL, VNM, HRV, ROU, HUN, BGR, SVN, PHL, IDN	ZAF
**87**	GRC, SVK, POL, VNM, LTU, EST, HRV, ROU, UKR, BGR, SVN, ARE, LVA	

## Conclusions and recommendations

This article proposes a model (LARDEG) from network science to reevaluate the world system/dependency (or core-periphery) theory of international hierarchy, structural inequality, and unbalanced trade between blocks. Accordingly, the network setup applied here contributes to filling the gap in the literature when discussing several thorny problems about the structural properties of the sectoral international trade networks: (1) At each hierarchical level, our procedure assigns each vertex either to a single ‘core’ set of vertices or to a single ‘periphery’ set of vertices. Taken together, our findings show that, unlike the first hierarchical level (where the ‘size effect’ is closely related to the core block membership), the second- or third-level ‘core’ relies so heavily on distance and geographical proximity. Moreover, closer analysis of differences between the GNI per capita of individual countries indicate that this indicator is a much poorer proxy for the world-system status. (2) The country’s position is highly correlated with its level of processing: high diversity in the types of products a certain country produces provides greater access to markets and trading partners. Hence, the question arises as to whether or not the structural positions remain unequal in terms of the level of processing?! Does the growth of export manufacturing in periphery really lead to dependency reversal and moving up the hierarchy? Or, do the changes associated with the global value chains simply result in different forms of dependence and reproduce global inequality? Network analysis conducted here permits a systematic and detailed investigation of the trade relationships within and between blocks. The various configurations of unbalanced trade between blocks may ultimately provide insights into the mechanisms of the observed patterns of the country mobility in the world economy. Overall, our results suggest that the rise of GVCs has recovered a vast and unequal global division of labor splitting the world into ‘headquarter’ economies located in the U.S., Japan, Germany and China, and the ‘factory’ economies placed in South-East Asia, Latin America and Eastern Europe. Tangible activities (e.g. assembly and processing) occur in ‘factory’ economies of developing countries providing labor, whilst intangible intellectual work (e.g. R & D and design) takes place in the ‘headquarter’ wealthy nations picking up the lion’s share of rents. That all seems to make perfect sense that in most developing countries exports have raised substantially without having led to comparable increases in domestic value-added, and thus dwindling the production-linked gains typically expected with export-led growth.

Do developing countries and latecomers to GVCs have the fate of being both eternal suppliers and locked into relatively low value-added activities? How can other countries repeat the journey of South Korea, Hong Kong or Singapore to promote local and international learning, catch up and leapfrog ahead into more value-added (complex) products, as well as to become more successful in dealing with ‘middle-income trap’, than for instance Indonesia or Brazil? Perhaps new methods from network science and complexity research [60–64], as well as further analysis of the value distribution (and chain ownership) can contribute to the challenge of unraveling the complex relations between global trade networks and income inequality. For instance, the core-periphery structure of sectoral international trade networks here has been observed with 3 algorithms, all designed for detecting blocks in graphs: one is our algorithm (LARDEG), while the other two are based on regular and stochastic equivalence. However, detailed analysis of trade networks with stochastic block-models (SBMs) and models beyond SBMs, for example, with models based on exchangeable random measures and edge exchangeable models [51], may be an important line of inquiry in future research. Moreover, our analysis is limited to international trade in goods (services are excluded). We do not know yet how digital technologies may change the international inequality associated with different types of goods. Finally, while this study was limited to assessing whether or not there were significant changes in the structure of economic relations between individual countries in the world economy as a whole, it may also be interesting to examine the disparities in regional performance, as well as the competition between hegemonic core states and potential rivals. Nevertheless, we believe that this paper, with its focus on measurement and interpretations of the structure and changes in the world-economy and global patterns, will contribute to a more complete political economy of the global system by providing an image of the structure and dynamics useful to other researchers.

## Supporting information

S1 File Basic data for the first-, second-, third-level core. (XLSX)

S2 File Industries the country appears at the first-level core blocks. (XLSX)

S3 File Average GNI_capita of core blocks at different levels. (XLSX)

S4 File Densities and trade flows at the first hierarchical level. (XLSX)

S5 File Inter-block mobility of countries. (XLSX)

S6 File ISO country codes and region. (XLSX)

S7 File Raw data. (ZIP)

S8 File Networks for each sector and year. (ZIP)
